# Resilience of refugees displaced in the developing world: a qualitative analysis of strengths and struggles of urban refugees in Nepal

**DOI:** 10.1186/1752-1505-5-20

**Published:** 2011-09-24

**Authors:** Fiona C Thomas, Bayard Roberts, Nagendra P Luitel, Nawaraj Upadhaya, Wietse A Tol

**Affiliations:** 1 Institute of Social Psychology, London School of Economics & Political Science, London, UK; 2 Faculty of Public Health and Policy, London School of Hygiene and Tropical Medicine, London, UK; 3 Transcultural Psychosocial Organization Nepal (TPO Nepal), Kathmandu, Nepal; 4 Global Health Initiative, Yale University, New Haven, USA; 5 HealthNet TPO, Amsterdam, the Netherlands

## Abstract

**Background:**

Mental health and psychosocial wellbeing are key concerns in displaced populations. Despite urban refugees constituting more than half of the world's refugees, minimal attention has been paid to their psychosocial wellbeing. The purpose of this study was to assess coping behaviour and aspects of resilience amongst refugees in Kathmandu, Nepal.

**Methods:**

This study examined the experiences of 16 Pakistani and 8 Somali urban refugees in Kathmandu, Nepal through in-depth individual interviews, focus groups, and Photovoice methodology. Such qualitative approaches enabled us to broadly discuss themes such as personal experiences of being a refugee in Kathmandu, perceived causes of psychosocial distress, and strategies and resources for coping. Thematic network analysis was used in this study to systematically interpret and code the data.

**Results:**

Our findings highlight that urban refugees' active coping efforts, notwithstanding significant adversity and resulting distress, are most frequently through primary relationships. Informed by Axel Honneth's theory on the *struggle for recognition*, findings suggest that coping is a function beyond the individual and involves the ability to negotiate recognition. This negotiation involves not only primary relationships, but also the legal order and other social networks such as family and friends. Honneth's work was used because of its emphasis on the importance of legal recognition and larger structural factors in facilitating daily coping.

**Conclusions:**

Understanding how urban refugees cope by negotiating access to various forms of recognition in the absence of legal-recognition will enable organisations working with them to leverage such strengths and develop relevant programmes. In particular, building on these existing resources will lead to culturally compelling and sustainable care for these populations.

## Background

Contrary to the iconic image of refugees in camps, about fifty percent of the world's 10.5 million refugees are classified as 'urban refugees' [1]. The number of urban refugees, that is, refugees from either an urban or rural background who have fled their home countries because of a fear of persecution and are now living in an urban area of a new country, are growing in comparison to camp-based refugees [2]. Looking for anonymity or landing in the city by chance, urban refugees face substantial and unique difficulties. Discrimination, unemployment, lack of housing and social support, and limited access to health services, as well as exposure to violence during and after flight are just some of the challenges urban refugees are confronted with in cities [2-4]. Such adversity may subsequently decrease their capacity to cope with acculturation stressors, potentially placing them at increased risk for mental illnesses [5]. Not only do urban refugees encounter challenges that are unique from those in refugee camps but by virtue of their origins, education and skill-set, they deserve to be handled differently from camp refugees [6].

Substantial literature has documented the mental health sequelae of torture, mass violence and forced migration for the displaced [5,7-9]. However, there is a paucity of literature on how urban refugees cope in their circumstances. Where studies do exist, they approach coping and mental health for refugees from a predominantly individualistic, biomedical perspective [5,10]. However, guidelines and research stress the importance of culture-informed knowledge to guide public mental health programmes [11,12]. For example, a study with urban refugees residing in Kampala found that social support from both the local population and other urban refugees as well as financial stability reinforced resilience [13]. Similarly, another study with urban refugees residing in Tanzania illustrated the importance of social-networks as a coping mechanism [14].

Research has highlighted the importance of recognising the resilience and agency of refugees and the need to better understand the different methods of coping with traumatic events and new and challenging circumstances of displacement [15-19]. As Almedom [20] notes, health and well-being go beyond the simple absence of disease and include the presence of capacity and conditions that promote wellness.

It has also been argued that the understanding of resilience and coping should not be approached from the individual level only [15]. In their work with Kenyan young carers, Skovdal et al [21], criticise the traditional understanding of coping as an individual undertaking and argue for coping as a function of the opportunities people have for engaging in positive forms of social participation. Such calls for a psychosocial approach have been recognised globally and are now included in leading international guidelines (e.g. IASC [11]; PWG [22]; 23).

Only recently emerging out of its own civil-conflict, Nepal has witnessed an influx of refugees from Tibet, Bhutan, and multiple surrounding countries [24]. In Kathmandu, there are approximately 300 urban refugees and asylum-seekers from 10 countries [25]. It is estimated that approximately half of the urban refugees in Kathmandu are Pakistani and about one-third are Somali; together, they constitute the two largest urban refugee populations in Nepal. This study focuses on these Pakistani and Somali refugees living in Kathmandu.

In their countries of origin, the *Ahmadiyya *Pakistanis were persecuted for their religious beliefs [26], while the Somalis feared for their lives because they belonged to minority tribes. Some Somalis were smuggled to Nepal with misguided hopes of ending up in Europe. Both groups are now in a country that is not a signatory to the 1951 Convention or the 1967 Protocol relating to the Status of Refugees and are stuck in a legal and political vacuum. In the absence of any formal framework offering protection for refugees in Nepal, the United Nations High Commissioner for Refugees (UNHCR) provides legal and physical protection for urban refugees [25]. However, urban refugees cannot obtain legal integration in Nepal, they cannot repatriate (i.e. return to their home country) unless they do so voluntarily, and third-country resettlement can take many years for the eligible or not happen at all for others. They are not allowed to legally work which exacerbates their already tenuous existence. With an already over-burdened healthcare system, there is little attention to mental health care among the general population, let alone refugees.

### Research Aims

The aims of this research were to explore the main challenges facing urban refugees in Kathmandu and to understand how they cope with their circumstances. Additionally, we also sought to explore how our findings fit within Honneth's theoretical framework (described below).

### Theoretical Framework

Axel Honneth's [27] work on *the Struggle for Recognition *is used as a theoretical framework to help guide and inform the study findings. Honneth's work was used as it emphasises the importance of legal recognition (for the purpose of this paper, we define legal-recognition as urban refugees being accorded the same rights as individuals in their host-country, in this case, Nepal) and larger structural factors in facilitating daily coping. Specifically, he presents an overlapping tripartite schema of *love*, *solidarity *and *rights *which enable the development of basic self-confidence, self-esteem and self-respect, respectively. For individuals to have status in society, the presence of these three elements is necessary. The extent to which urban refugees experience these elements can enable or inhibit coping capacity.

Following Honneth's conception, love is understood here as "successful affectional bonds to other people" (p.104). For Honneth *love *forms the precondition for basic self-confidence. Such self-confidence is founded upon primary-relationships consisting of strong emotional ties among a select group. Individuals are tied to the existence of others who reciprocate one's positive self-valuation resulting in trust in oneself and self-confidence.

With regard to our research aim on the fit of Honneth's theoretical framework, we include religion under love. Although religion is not discussed in Honneth's work, it was frequently mentioned by our participants as an important coping strategy in providing emotional support, similar to that provided by primary-relationships associated with Honneth's element of *love*.

*Solidarity *is premised on social appreciation, leading to the development of self-esteem. Here, the emphasis is on people's unique attributes which are not shared with others. Accordingly, one must feel that they have something valuable to contribute as having nothing of value to offer impedes the development of one's unique skills and identity. *Rights *and self-respect are inextricably linked in that rights enable one to raise and defend claims illustrating to the individual that he/she is legitimately respected by others [28]. To facilitate the possibility of making claims on equal terms with others, recognition of an individual as an autonomous legal person must be accorded to each subject equally [29]. While a person without rights can certainly have self-respect, Honneth argues that the fullest form of self-respect is only attained through legal-recognition.

**Figure 1:**
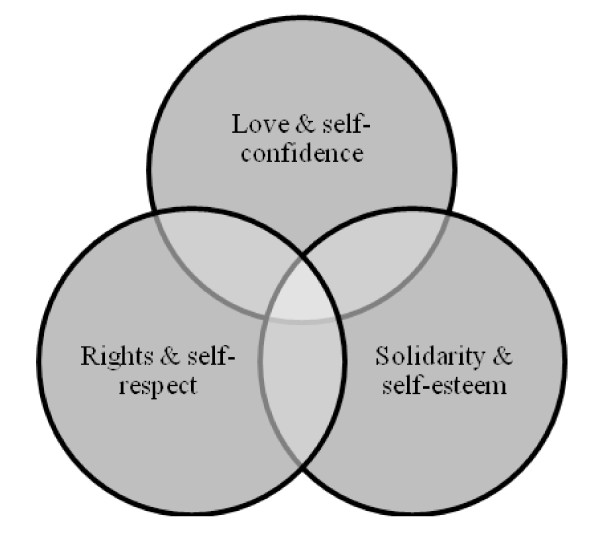
**Visual depiction of Honneth's theory on love, solidarity & rights**.

Moreover, there must be respect for the citizens' rights in practice. Thus, an agent's capacity to raise and defend claims can form the basis of self-respect only if this capacity can actually be exercised; such opportunity for participation however, can only be taken advantage of if individuals have a certain social standard of living, which includes a minimum of cultural education and economic security. Honneth's theory, based on the intersubjectivity of individuals, sets the backdrop for understanding coping as a function that exists beyond the individual. An individual's relationship to self is not a solitary development but an intersubjective process in which one's perspective emerges through interactions with others perspectives; it follows then that when any of the intersubjective processes are denigrated, the means to coping will likewise be intersubjective and a product of the community. Figure 1 provides a depiction of the inter-subjectivity of the elements in Honneth's theory.

In the present context, this type of framework is intended to guide the research and inform the research findings. Such research may subsequently contribute to developing effective programmes to address the needs of urban refugees.

## Methods

As little is known about the coping mechanisms of urban refugees in Kathmandu, we felt an open-ended exploratory inquiry would be most conducive to understanding their circumstances. Specifically, focus groups and semi-structured interviews were used. Focus groups provided a genuine setting for social interaction while the individual interviews enabled participants to discuss the highly personal experience of displacement that may at times be challenging or painful to speak about.

The interview methods were supported by the use of Photovoice methodology. This is a participatory action research method where participants take photographs relating to their life experiences and beliefs on a certain topic. This provides a valuable means to help participants express and discuss their experiences and beliefs [30,31]. Photovoice provided visual examples to prompt and facilitate the narrative accounts given by participants.

### Study participants and setting

The study population was Pakistani and Somali refugees (described above) who live in Kathmandu. They generally live clustered together and reside in cramped houses and apartments, and most have lived in Kathmandu for at least three years. Criteria for participation included being a Somali or Pakistani urban refugee in Kathmandu aged above 18-years. Adults (aged 18 and above) were recruited because it was felt the experiences and coping strategies for children would be very different to adults and so required separate research, which was beyond the scope of this study. While urban refugees from Afghanistan, Myanmar, Sri Lanka, Iraq and other countries reside in Kathmandu, this study focused on Somali and Pakistani urban refugees because they represent two population groups likely to have very different experiences, but with relatively homogenous experiences within groups. Also, as the two largest urban refugee populations in Kathmandu, we were interested in providing a study with policy relevance. Other than country of origin, minimum age and gender mix, there were no specific criteria and instead we preferred an open sampling approach through the use of convenience and snowball sampling.

There were 24 participants: 16 Pakistanis and 8 Somalis. We sampled for more Pakistani participants as the number of registered urban refugee Pakistanis in Kathmandu is almost double that of registered Somalis. Fifteen men and nine women participated. Pakistani participants ranged in age from 23-47 years while Somali participants were 18-52 years old (Table 1). While we did not conduct separate analysis for different age groups, some unique findings arose for those under the age of 30. As such, where relevant, we identify this group as 'youth and young adults' in our results.

**Table 1:** Participant demographics

Code	Gender	Age	Interview type	Background	Interview language
P1	M	39	Interview	Ahmadiyya Pakistani	Hindi
P2	M	23	Interview/FG	Ahmadiyya Pakistani	Nepali
P3	M	30	FG	Ahmadiyya Pakistani	Hindi
P4	M	34	FG	Ahmadiyya Pakistani	Hindi
P5	M	35	FG	Ahmadiyya Pakistani	Hindi
P6	M	37	FG	Ahmadiyya Pakistani	Hindi
P7	M	33	FG	Ahmadiyya Pakistani	Hindi
P8	M	24	Interview	Ahmadiyya Pakistani	Hindi
P9	M	47	Interview	Ahmadiyya Pakistani	Hindi
P10	M	23	Interview/FG	Ahmadiyya Pakistani	Hindi
P11	M	24	Interview	Ahmadiyya Pakistani	Hindi
P12	M	35	Interview	Ahmadiyya Pakistani	Hindi
P13	F	31	Interview	Ahmadiyya Pakistani	Hindi
P14	F	36	Interview	Ahmadiyya Pakistani	Hindi
P15	F	35	Interview	Ahmadiyya Pakistani	Hindi
P16	F	31	Interview	Ahmadiyya Pakistani	Hindi
P17	M	36	Interview	Somali	English
P18	F	20	FG	Somali	Somali
P19	F	38	FG	Somali	Somali
P20	F	30	FG	Somali	Somali
P21	F	20	Interview/FG	Somali	English/Somali
P22	F	52	FG	Somali	Somali
P23	M	18	FG	Somali	Somali
P24	M	29	Interview	Somali	English

Through the assistance of Transcultural Psychosocial Organisation-Nepal (TPO-Nepal) and UNHCR's implementing partner in Kathmandu, Propublic, participants were recruited using convenience and snowball sampling [32]. This was done through an advertisement posted at Propublic's community centre and through word-of-mouth. Interested participants were asked to sign-up with a social worker at the community centre, during which time we also asked participants to inform others in their community about the study. Participants were given disposable cameras for the Photovoice activity one week before the focus groups and interviews and were asked to take ten pictures of people and things that help them overcome and deal with difficult situations. Two focus groups were then held, both of which included verbal narratives about the photographs. One focus group was conducted with six Pakistani men (as Pakistani refugees felt it was not appropriate to have a discussion with women and men together) and another with six Somalis (five females; one male). Few Pakistani women signed-up for the group discussion and so individual semi-structured interviews were conducted with them instead. The Pakistani focus group included participants aged between 30-37 years old with one 23 year old while the Somali focus group consisted of participants aged 18-52. These age ranges are typical of Pakistani and Somali urban refugees in Kathmandu. Fourteen semi-structured individual interviews were also held with women and men from the Somali and Pakistani refugee communities.

The discussions and interviews were all conducted in April 2010 in Kathmandu, Nepal. The majority of the interviews and the Pakistani male focus group were conducted in the UNHCR/Propublic-run community centre; a familiar and convenient location for the participants. A select number of interviews were conducted in participants' homes at their request, and a number of interviews and the Somali focus group were conducted in the TPO-Nepal office.

The interview topic guide broadly covered personal experiences of being a refugee in Kathmandu, perceived causes of psychosocial distress, and strategies and resources for coping. It was designed based on a preliminary literature review and revised after feedback from various experts in the field of mental health and research staff at TPO-Nepal. The focus groups were supplemented by the initial discussions from the Photovoice exercise. The focus group participants shared the photographs amongst themselves and explained why they selected certain photographs and what they represented about stressors and the person/thing photographed that helped them cope.

The interviews and focus groups with the Pakistani participants were conducted in Hindi as the participants, facilitator and the first author (FCT) all spoke Hindi. For the interviews and focus group with Somalis, the lead researcher (FCT) asked the questions in English and this was translated by the translator into Somali. The translator briefly summarised responses for FCT so that she was able to ask follow-up questions that were tangential to the topic guide. With permission from the participants, all but one interview was digitally-recorded. The interviews were transcribed and translated by the facilitators.

### Analysis

Thematic network analysis was used in the study [33]. A systematic and iterative process was followed to interpret and code the data. This consisted of several stages: first, transcripts were read to gain familiarity with the data; second, relevant text segments from the transcripts were condensed into brief words or phrases known as codes; third, basic themes were derived from exploring the various issues discussed within the coded segments; fourth, basic themes were grouped under the broader themes of vulnerability and Axel Honneth's schema of *love*, *rights*, and *solidarity *(see above). As illustrated in the results, the inability to work came up frequently. The legal prohibition to working was coded under *rights*. However, the social and personal consequences of not working (e.g. the ability or inability to utilize one's skills and resulting self-esteem), were coded under *solidarity*. Analysis was not conducted on the photographs taken during Photovoice, but narratives related to the pictures were included in the analysis.

### Ethical procedures

The study was granted ethical clearance by the Institute of Social Psychology Research Ethics Board at the London School of Economics. This study was conducted in collaboration with TPO-Nepal and Propublic, partners of UNHCR Nepal. UNHCR Nepal was informed of the interviews for the purpose of receiving recommendations. Written, informed consent was obtained from all participants prior to conducting the interviews and focus groups. As the nature of the discussion was sensitive at times, a counsellor was on call during interviews to provide assistance (this was never needed).

## Results

This section discusses the various mechanisms of coping using Honneth's schema of *love *(self-confidence); coping through *solidarity *(self-esteem) where possible; and the inability to cope through *rights *(self-respect). To help understand these mechanisms, it is first necessary to grasp the context within which their vulnerabilities are experienced.

### Perceived vulnerabilities

Vulnerability was characterised by discrimination, daily stressors, unfulfilled expectations, and lack of control, culminating in generally poor reported mental health.

Discrimination is a stressor that has followed both groups from pre-displacement to their current location. It is because of discrimination that both groups fled their homelands, yet in Nepal, they continue to live in fear of abuse as a result of their religious beliefs or skin colour. As a Pakistani male (age 47) participant articulated:

P9: They are trying to label us as terrorists. They don't even treat us like humans. We are treated like this just because we are Muslims. Pakistan does not consider us as Muslims and other countries behave with us badly because we *are *[emphasis added] Muslims...We are in crisis from both the sides.

For Somalis, the challenge is two-fold. Like the Pakistanis, they are discriminated against because of religion. However, it is their visible differences that put them at increased risk.

P18: It is even difficult to roam on the streets, people start staring at you, they will start calling you names like *kala*, *habsi *['black' in derogatory tone] (Somali female, 20).

Discrimination infused multiple aspects such as searching for housing to shopping in markets. Amidst this discrimination, it is important to note that some refugees spoke of the kindness of their landlords or their slow integration with the Nepali populations. These instances, however, were experienced by a minority of participants. For most interviewees, there exists a discord between how they imagined their lives and how their reality has manifested, revealing a common thread of unfulfilled expectations. Seeking refuge, many envisioned a better life that included more than just protection.

Compounding the discrimination urban refugees face and the weight of their unmet expectations, are the daily stressors they encounter. A frequently mentioned issue was that of finding and keeping housing, financial dependence on UNHCR assistance, and lack of employment:

P22: The Nepalese don't want to rent us their flats because of our colour.....From the morning till evening we go out to look for a house everywhere in the city. Even with the help of the Somali community it was difficult for us to find a house (Somali female, 52: focus group).

Separation from family left behind was another predominant issue for both groups:

P9: We don't have tension regarding food but when we eat we remember our past. I think about what my children must be eating and what they must be doing (Pakistani male, 47).

In addition, many felt they had little control over the direction of their lives. Lack of control in their present day was frequently reflected in fatalistic attitudes:

P24: People used to be so desperate but I think now they are all disappointed. They used to protest to the government but I think they have all surrendered. They're tired. Let's wait for things to happen instead of making it happen (Somali male, 29).

Respondents also reflected on the challenges to maintaining mental well-being under such circumstances:

P20: When we are at home alone, it is all about thinking, painful thoughts. Sometimes you cannot sleep because of those thoughts...wondering what the future holds for you (Somali female, 30: focus group).

### Love as a pre-requisite for self-confidence

In Nepal, affectionate bonds amongst urban refugees were largely constructed through relationships with supportive friends and family members. These relationships functioned as a mode of resilience for many. They provided a buffer against the vulnerabilities mentioned in the previous section and reduced anxiety through psychological support. Religion also played a similar role in people's lives and functioned as a significant coping mechanism. Coping through others was raised by both groups:

P12: Our community has been living like a family. Everyone comes together whenever anyone faces trouble. This helps to lessen the tension...I think we have been able to live here in this condition for this long because of this system of helping each other (Pakistani male, 35).

P20: We are happy when we come together - Somalis as a group when we come together - when we come to the community centre, when we come together in our homes, when someone is sick and we come together, we feel happy (Somali female, 30: focus group).

Respondents' children were repeatedly mentioned as a significant source of support, and the majority of photographs taken during the Photovoice exercise were those of participants' children. Parenthood helps to counteract the everyday psychological insecurity individuals face.

Friendships went beyond providing psychological support and reducing anxiety during trying times; they also functioned as a motivator for improving skills where possible. For example, one participant spoke of his friend who taught him English by watching football matches and using a Somali-English dictionary to translate words they did not know. Solace, and subsequently, a relationship of recognition were thus found through the existence of others who reciprocated feelings of esteem [27].

Many respondents mentioned the respite they found through the cathartic process of prayer, including how religion and God acted as a buffer for the thoughts of suicide:

P9: Sometimes I have thoughts of committing suicide. How long can a person live with such problems? Then, I look at the sky and remember him [indicating to God]. He is the one who gave us life so we will die by his will (Pakistani male, 47).

### Solidarity as a pre-requisite for self-esteem

As Honneth argues, self-esteem develops when one distinguishes oneself from others based on having valuable traits or characteristics to offer. In this way, there is the opportunity to develop one's identity through his/her individuality, resulting in self-esteem. In the absence of legal employment for urban refugees in Kathmandu, limited opportunities existed within which to develop and express one's unique skills and traits.

Several participants spoke of their frustrations regarding their inability to legally work. Beyond leading to precarious financial circumstances, the inability to work meant, for many, the inability to use well-developed skills or to develop a new skill-set:

P1: Right now we are living the lives of a beggar. We are living on an allowance that we are given which is like alms to us. We do not like to take allowance but we have no choice. We wish we had a job and could earn and use our skills...we used to be self-sufficient people (Pakistani male, 39).

This desire, and consequent frustration from an inability to manifest it, was especially apparent amongst youth and young adults:

P24: For those who came here at a very young age - 16, 17 - a very important part of their youth is taken away and they can't do anything about it. That is what makes people so frustrated. For those with small children, they go to school...but for the others, there is nothing.

An additional frustration was the issue of realising one's skills but not having the opportunity to exercise it. One participant mentioned:

P8: I have learnt computer software and photo-shop. I do not know when it is going to yield results (Pakistani male, 24).

While there is access to primary education for youth, young adults expressed feeling limited in terms of opportunities to express their unique attributes in the absence of meaningful activities and peer networks:

P10: Every person has their own will. They have their own thoughts. I wish I was given some authority to work and contribute (Pakistani male, 23).

Although limited, when opportunities for self-realisation presented themselves they enabled people to feel they had something valuable to offer:

P16: I was very happy when the community centre opened. I used to feel very lucky and I thought to work as a volunteer. I didn't even think much about settling down anywhere else. The staff members of UNHCR also gave me the opportunity to work in the programme for children...I made different things for the children and parents in the winter camp. I was very excited (Pakistani female, 31).

Others showed appreciation for the opportunities the community centre offered:

P23: My frustrations are decreasing. Now there's a computer class, there could be another one for mechanics, so if they start that education class, we may be happy about it (Somali male, 18: focus group).

Another avenue through which some, especially male youth, found the potential for self-realisation was through engaging in sports. One participant repeatedly mentioned his passion for playing football and noted how it acted as a coping mechanism. This sentiment was echoed by other male youth who were interviewed. Another participant mentioned how his skills in homeopathy helped him feel good by taking care of his community members:

P9: I pass my time by doing homeopathy...When a patient comes to me for treatment and tells me about his painful story I forget about mine because I feel good helping him (Pakistani male, 47).

### Rights as a pre-requisite for self-respect

When available, the two elements of love and solidarity facilitated respondents' coping ability and helped maintain basic self-confidence and self-esteem. Lacking however, was the ability of urban refugees to maintain full self-respect as obtained through legal-recognition; this systematic failure consequently impedes full coping capacity.

The absence of legal recognition translates into multiple limitations in the lives of urban refugees, including the aforementioned inability to legally work;

P1: We are not allowed to work here. It is difficult to pass the time doing nothing all day. When we stop working our hands and mind also stop working. We feel much tensed when we have a lot of leisure (Pakistani male, 39).

Many mentioned resorting to illegal (labour) work for the sole purpose of generating additional income. Such work was largely possible only for the Pakistanis. Even if Somalis wanted to work illegally, their physical differences from the local population prevented them from doing so as they would be at increased risk of getting caught by the authorities.

A general sense of feeling 'stuck' pervaded discussions. From the three potential options of repatriation, legal-recognition in Nepal or third-country resettlement, few participants felt these were realistic scenarios in the near future. While many participants could arguably be expected to have substantial esteem acquired through their valuable and unique skills, such esteem was insufficient without the opportunity for further recognition.

Many urban refugees attempted to assert their claims either through protesting or other collective means. However, as the dialogue below from the Somali focus group shows, there was frequent interference in their sphere of liberty when urban refugees attempted to exercise their claims:

P20: We were protesting to both sides until they called the police on us, and the police were not even respecting us as women (female, 30).

P22: for 40 days we were sleeping outside (female, 52)

P20: we did whatever we could. Even some of our youth made a hunger strike.

P19: I was one of those who made the hunger strike. The police arrested us and we were in custody for 10 hours. We didn't even do anything - they accused us of blocking the road but we didn't (female, 38).

## Discussion

This study was framed by our interest in the resilience of urban refugees - a group increasing in number, but receiving little research attention - and influenced by our experience of individuals as active social actors rather than passive victims of circumstances beyond their control [16,17]. Approaching the study of urban refugees with Honneth's theory on recognition enables us to understand the different types of recognition that facilitate coping and build resilience among those urban refugees who access the social and symbolic resources to help them cope. While Honneth has been criticised for being too abstract in his meaning of recognition [29] using his work as a theoretical-framework nonetheless provides useful pointers to the different social-psychological resources that facilitate/impede coping strategies, which can have implications for policy and practice.

The findings from this study also illustrate Honneth's elements of love, solidarity and rights leading to self-confidence, self-esteem and self-respect, respectively. The integral role of family, friendship and religion provided the emotional support found in *love *thus enabling the maintenance of a basic level of confidence. Likewise, social appreciation enabled individualisation and thus resulted in a feeling of solidarity within a community of value.

However, as an exploratory study with convenience samples, care must be taken in interpreting the findings outlined in this paper, especially because of the small sample size and the specific location of the study. Participants were sampled through the UNHCR/Propublic community centre and this may have biased the sample. There are likely to be many urban refugees who come to the centre infrequently and may not have heard about this study. To circumvent this issue, we asked participants to spread the word so that others, who were not present at the time, could learn about the study. We also faced difficulty in recruiting sufficient Pakistani women. Finally, while some degree of saturation was reached with these numbers, further interviews with Somali and Pakistani urban refugees as well as urban refugees from other populations could shed more light on their circumstances.

Notwithstanding these limitations, the study findings illustrate that even in a legal and political vacuum of formal non-existence, refugees in our sample found the ability to give meaning to their lives. As Muecke [15] notes, "...refugees present perhaps the maximum example of the human capacity to survive despite the greatest of losses and assaults on human identity and dignity". To understand which specific factors facilitate coping, we drew upon Honneth's theory of recognition. This suggests that for coping strategies to develop, mutual recognition from primary-relations, social-networks and the legal order, need to be present. In this study, participants coped with the absence of legal-recognition by drawing upon recognition from the former two. Specifically, in the void of institutional recognition, participants turned to obtaining recognition primarily through their immediate networks (*love) *and where available, through avenues to contribute something valuable to a larger project (*solidarity)*. While *love *and *solidarity *provided some recognition, full recognition was hampered in the absence of legal recognition.

Support from close relations was frequently mentioned as a coping strategy. This resonates with findings in studies of refugees in Tanzania [14], South Africa [34], and Western Europe [35]. Such primary relations functioned as a mechanism of attaining basic self-confidence and contributed to daily coping capacity. This is not to imply that those with close networks do not experience difficulty in Kathmandu, but that obstacles are met with greater ease where one receives validating recognition from others. For example, many participants mentioned that shortly after arriving in Kathmandu they immediately searched for others from their community to help with acculturation in their foreign surroundings.

Although not mentioned by Honneth, we include religion as an element of *love*. As a coping strategy frequently reiterated by participants, it provided meaning to life circumstances, helped develop self-confidence, and played a key role in how participants coped with adversity. Belief in God does not necessarily result in the type of positive reinforcement seen in primary-relationships, but emotional support was frequently derived through prayer and belief in a higher being. While religion is frequently mentioned as a coping mechanism in this study as well as with other refugee populations [36], it does not always fit well in western frameworks of coping, including Honneth's. Honneth's theory discusses the importance of close relations but does not shed light on the resilience religion builds for many. In this way, the findings on religion challenge Honneth's model and point to the need for amendment in the context of this study population.

Whilst the presence of a close-network strengthened coping capacity and the absence of legal-recognition limited it, this stark duality was less clear when it came to solidarity. There was almost a unanimous *desire *to 'do something' and use skills and contribute valuably in some way. However, such opportunities presented themselves infrequently. When the opportunity to contribute was made available, many participated and spoke of the value they derived from the experience. From personal observations at the community centre, we saw urban refugees teaching computer and English classes, fixing furniture, translating and participating in other such activities that exhibited each of their unique capabilities. With an omnipresence of *love *amongst urban refugees and the long road to achieving some form of legal-recognition, *solidarity *represents a challenging, yet effective coping mechanism to leverage. Many urban refugees have insightful suggestions regarding how their needs could be met, including how to leverage aspects of *love *and *solidarity*; these suggestions should be listened to and integrated wholeheartedly.

Such findings resonate with a separate body of literature on refugee integration. While this literature is in reference to refugees settling in mostly developed contexts, the settings tend to be urban and explore many similar issues which emerged from the data of the present study. For example, Strang and Ager discuss the value of employment [37] and social capital [38] in restoring self-esteem and enabling self-reliance as two of many factors in facilitating refugee integration.

Urban refugees have the potential to uniquely contribute to their social context [6,37]. They may bring with them new or different skills, knowledge of markets in their home countries, and useful business experience; for instance, self-settled refugees in Nairobi were found running their own businesses, many of which employed local Kenyans and refugees [17]. The respondents in our study bring with them a range of knowledge and skills and opportunities need to be made available for them to exercise and share the above.

In Kathmandu, some opportunities have presented themselves, albeit usually in the form of teaching or providing translation services for UNHCR and for those receiving psychosocial services through TPO-Nepal. The challenge to providing further opportunities lies in recruiting urban refugees and having their qualifications legitimately recognized as many urban refugees lack proper documentation. In such circumstances creative ways of recognising such talents and skills should be realised. As Jacobsen [39] notes, appropriate programmes can help host-states realise the potential of refugee resources and meet their demand for self-realisation in the forms of education, training and employment.

Such programmes however, should address the unique circumstances of different urban refugee groups. In our study, the aforementioned role of *love *and *solidarity *in facilitating coping was common to both Pakistani and Somali urban refugees in Kathmandu. Participants from both groups found solace through others and through their religious beliefs and many wanted the opportunity to contribute and develop their unique skills. Yet, in some instances, circumstances were unique to each group. For example, while both Pakistani and Somali participants spoke of encountering discrimination, as mentioned in our findings, Somalis noted the additional barriers they faced because of their visible differences to the local population. For Pakistani participants, many reported relatively high levels of education and subsequent frustration with the lack of programmes that met their skill level. Such differences between groups present varying implications for the uptake and sustainability of different programmes for urban refugees in Nepal. Unique challenges may also exist for other urban refugees in Nepal and these should be explored further.

In Kathmandu, urban refugee children have access to primary-education through UNHCR funding. However, as illustrated in the findings, youth and young-adults long for, and lack access to more education. Programmes, be they vocational training, sports activities, youth clubs or further education, could be beneficial for this portion of the population. While the importance of individualised therapy should not be underestimated, as Miller and Rasmussen [40] note, a narrow psychosocial focus runs the risk of underestimating the need for specialised treatment for depressed or persistently traumatised individuals. In this way, programmes that seek to develop esteem and build resilience should be developed alongside individualised therapy for those who find it valuable.

Ultimately, what is needed is something more sustainable than just transient opportunities. Providing refugees with critical opportunities will facilitate self-reliance in their current circumstances as well as in the event of a durable solution. Indeed, under the right conditions, the skills of urban refugees will enable them to not only become self-sufficient but to be beneficial to their host society. Parallel to this, it is crucial for organisations to maintain advocacy with the government of Nepal in signing the relevant conventions. Unless legal recognition is afforded to urban refugees in Nepal, full coping capacity will remain hampered.

## Conclusions

In this paper, we have sought to emphasise the importance of recognition in understanding and promoting coping and resilience amongst urban refugees. To do so, we focused on urban refugees in a unique context of non-recognition. While this research has focused on the resilience of urban refugees, multiple participants spoke of their vulnerabilities. It is not our intention to overshadow the bleak reality that many of the participants face. Instead, focusing on the few positive instances in the midst of uncertainty is a purposeful attempt to promote research and policy-setting that supports resilience and coping among urban refugees. Ultimately, programme and research interventions need to enhance existing coping abilities by acknowledging urban refugees as agents. This does not mean that focusing on the agency of urban refugees detracts responsibility from the host government or from other agencies. External support is vital but should be designed in a way that builds resilience and facilitates coping.

## Competing interests

The authors declare that they have no competing interests.

## Authors' contributions

FCT conceived, designed and coordinated the study, collected the data, and drafted the manuscript. BR participated in the study design and assisted in the refinement of the theoretical framework. NPL and NU contributed to the design and coordination of the study and assisted with supervising the research group. WAT contributed to the design and coordination of the study, supervised the research group, and assisted with analysis of text and refinement of theoretical framework. All authors read and approved the final manuscript.
